# *RTN1*在肺腺癌中表达及对免疫微环境的影响

**DOI:** 10.3779/j.issn.1009-3419.2022.105.02

**Published:** 2022-06-20

**Authors:** 帅 朱, 玲玲 祖, 嵩 徐

**Affiliations:** 300052 天津，天津医科大学总医院肺部肿瘤外科，天津市肺癌研究所，天津市肺癌转移与肿瘤微环境实验室 Department of Lung Cancer Surgery; Tianjin Key Laboratory of Lung Cancer Metastasis and Tumor Microenvironment, Lung Cancer Institute, Tianjin Medical University General Hospital, 300052 Tianjin, China

**Keywords:** 肺腺癌, 免疫治疗, RTN1, 预后, 免疫微环境, Lung adenocarcinoma, Immunotherapy, RTN1, Prognosis, Immune microenvironment

## Abstract

**背景与目的:**

网状体家族基因1(Reticulon family gene 1, *RTN1*)是一种与内质网相关的网状体编码基因。RTN1在神经内分泌细胞的膜运输或神经内分泌分泌中起关键作用，同时RTN1可作为有神经内分泌成分的恶性肿瘤的潜在诊断/治疗标志物。然而在肺腺癌(lung adenocarcinoma, LUAD)患者中RTN1的表达情况及其对免疫微环境影响均未有报道。本研究旨在使用公共数据库和生物信息学网络工具研究RTN1在肺腺癌中的表达及其与肺腺癌中免疫浸润和存活的相关性。

**方法:**

使用Tumor Immune Estimation Resource 2.0(TIMER 2.0)和Gene Expression Profiling Interactive Analysis 2(GEPIA 2)分析肿瘤和正常组织中RTN1 mRNA的表达水平。使用人类蛋白质图谱检查RTN1蛋白质表达。利用GEPIA2在线工具分析癌症基因组图谱(The Cancer Genome Atlas, TCGA)中RTN1的临床预后意义。为了进一步确认RTN1的潜在功能，使用基因集富集分析对数据进行了分析。此外，我们对来自肿瘤免疫单细胞中心(Tumor Immune Single-cell Hub, TISCH)数据库的两个数据集在单细胞测序水平上进行降维聚类分析，观察RTN1在不同种类免疫细胞的细胞聚类。使用TIMER在线工具分析预测TCGA队列肺腺癌患者免疫微环境中不同种类免疫细胞浸润丰度；使用TIMER和CIBERSORT研究与RTN1共表达基因和其相关肿瘤浸润免疫细胞之间的关系；最后，使用TIMER分析RTN1与免疫检查点之间的相关性。

**结果:**

我们发现LUAD患者的RTN1表达降低，且与患者的预后密切相关。RTN1参与吞噬体的形成、造血细胞形成和细胞黏附的过程，对T细胞活化起着重要作用，使用cBioPortal与TCGA数据来分析，发现*RTN1*与*BTK*、*CD4*、*ECSF1R*、*MNDA*、*NCKAP1L*和*SNX20*显著相关。以上基因高表达可能会引起CD4^+^ T细胞、肥大细胞、单核细胞、髓样树突状细胞和M1型巨噬细胞显著上调。并且，RTN1表达与常见免疫检查点CD274、CTLA4、HAVCR2、LAG3、PDCD1、PDCD1LG2、TIGIT、SIGLEC15密切相关。

**结论:**

RTN1作为一种肿瘤抑制基因并可能与更好的预后相关。此外，RTN1与可能参与免疫治疗反应的免疫浸润有关。但与其相关的机制需要进一步的研究。

## Abstract (en)

**Background and objective:**

Reticulosome family gene 1 (*RTN1*) is a reticulosome-encoding gene associated with the endoplasmic reticulum. RTN1 plays a key role in membrane trafficking or neuroendocrine secretion of neuroendocrine cells, while RTN1 serves as a potential diagnostic/therapeutic marker for neurological diseases and cancer. However, the expression of RTN1 and its effect on the immune microenvironment in patients with lung adenocarcinoma have not been reported. In this study, we aimed to investigate the expression of RTN1 in lung adenocarcinoma and its correlation with immune infiltration and survival in lung adenocarcinoma using public databases and bioinformatics network tools.

**Methods:**

Expression levels of RTN1 mRNA in tumor and normal tissues were analyzed using Tumor Immune Estimation Resource 2.0 (TIMER 2.0) and Gene Expression Profiling Interactive Analysis 2 (GEPIA 2). RTN1 protein expression was examined using the Human Protein Atlas. The clinical prognostic significance of RTN1 was analyzed using the GEPIA2 plotter database. To further confirm the potential function of RTN1, the data were analyzed using gene set enrichment analysis. In addition, We performed dimensionality-reduced clustering analysis at the single-cell sequencing level on two datasets from the Tumor Immune Single-cell Hub (TISCH) database to observe the cellular clustering of RTN1 in different types of immune cells. Using the TIMER online tool to analyze and predict the infiltration abundance of different types of immune cells in the immune microenvironment of lung adenocarcinoma patients in the TCGA cohort; TIMER and CIBERSORT were used to study the relationship between genes co-expressed with RTN1 and its associated tumor-infiltrating immune cells; finally, TIMER was used to analyze the relationship between RTN1 and immune correlations between immune checkpoints.

**Results:**

We found that RTN1 expression was decreased in patients with lung adenocarcinoma and was closely related to patient prognosis. RTN1 is involved in the process of phagosome formation, hematopoietic cell formation and cell adhesion, and plays an important role in T cell activation. Using cBioPortal and TCGA data to analyze, it is found that *RTN1* is significantly associated with *BTK*, *CD4*, *ECSF1R*, *MNDA*, *NCKAP1L* and *SNX20*. High expression of the above genes may cause significant upregulation of CD4^+^ T cells, mast cells, monocytes, myeloid dendritic cells and M1 macrophages. The expression of RTN1 is closely related to the common immune checkpoints CD274, CTLA4, HAVCR2, LAG3, PDCD1, PDCD1LG2, TIGIT and SIGLEC15 immune checkpoints.

**Conclusion:**

*RTN1* may act as a tumor suppressor gene and indicate better prognosis. Furthermore, RTN1 is associated with immune infiltration that may be involved in the immunotherapy response in LUAD. However, the related mechanism needs further research.

有数据^[1]^表明，肺癌是全球第二最常见的癌症，但是肺癌导致的死亡依然占据最大的比例。84%的肺癌是非小细胞肺癌(non-small cell lung cancer, NSCLC)，确诊的肺癌中只有20%-30%可以通过根治性手术治疗^[2]^。免疫检查点抑制剂，如程序性死亡受体-1(programmed cell death protein 1, PD-1)、程序性死亡配体-1(programmed cell death protein ligand 1, PD-L1)和细胞毒性T淋巴细胞相关抗原4(cytotoxic T-lymphocyte-associated protein 4, CTLA-4)抑制剂，已在黑色素瘤、肾癌、NSCLC和其他肿瘤中显示出良好的疗效^[3-5]^。尽管免疫检查点抑制剂在肺腺癌患者中有显著的临床疗效和持久的应答，但抗PD-1/PD-L1治疗在肺腺癌患者群体中的总有效率仅为20%-30%^[6]^。因此，迫切需要寻找其他有利于预测免疫治疗对肺腺癌患者的生物标记物以提高免疫治疗有效率，包括肿瘤细胞相关生物标记物、肿瘤免疫微环境(tumor microenviroment, TME)相关生物标记物、液体活检相关生物标记物和宿主相关标记物。

网状体家族基因1(Reticulon family gene 1, *RTN1*)(以前称为“神经内分泌特异性蛋白”)是一种与内质网相关的网状体编码基因。网状体在神经内分泌细胞的膜运输或神经内分泌分泌中起关键作用。RTN1编码3个变体，即NSP-A、NSP-B和NSP-C，它们通过同源C末端区域中的2个假定跨膜结构域连接到内质网^[7-10]^。

先前的研究^[11, 12]^已将RTN1作为神经系统疾病和癌症的潜在诊断/治疗标志物引入。RTN1被认为是具有神经内分泌特征的癌症的潜在标记物^[8]^。已有研究^[11]^表明，RTN1可降低BCL2样蛋白1(BCL2L1)编码的一种蛋白[即B细胞淋巴瘤-特大型(BCL-XL)]的抗凋亡活性。事实上，RTN1可以改变此蛋白的亚细胞定位，从线粒体到内质网，从而破坏其抗细胞凋亡的作用。

然而在肺腺癌患者中RTN1的表达情况及其对免疫微环境影响均未有报道。在本研究中，我们旨在使用公共数据库和生物信息学网络工具研究RTN1在肺癌中的表达及其与肺癌中免疫浸润和存活的相关性。这些发现或许将有助于确定肺癌预后的潜在生物标志物，并有助于个体化免疫治疗。

## 1 材料与方法

### 1.1 The Cancer Genome Atlas(TCGA)数据库

TCGA(https://www.cancer.gov/tcga)是一个公共资助的项目，旨在通过大规模基因组测序，在30多个人类肿瘤的大型队列中编目和发现主要的致癌基因组变化^[13]^。TCGA包括RNA测序、miRNA测序、DNA测序、DNA甲基化测序和临床信息。从TCGA下载肺腺癌(lung adenocarcinoma, LUAD)患者的转录组和临床资料，通过整理获得516例患者的表达矩阵和临床信息。

### 1.2 GEPIA 2数据库分析

GEPIA 2(http://gepia2.cancer-pku.cn/)是基因表达谱交互分析的更新版，用于分析来自TCGA和GTEx程序的9, 736个肿瘤和8, 587个正常样本的RNA测序表达数据^[14, 15]^。进行肿瘤/正常组织差异表达分析、患者生存分析、相似基因检测、相关分析和降维分析。用GEPIA2软件分析LUAD患者RTN1表达与TCGA数据和GTEx数据匹配的差异，并分析RTN1的预后意义。

### 1.3 TIMER数据库分析

TIMER是用于系统分析不同癌症类型的免疫浸润的综合资源(https://cistrome.shinyapps.io/timer/)TIMER应用先前发表的统计方法来从基因表达谱推断肿瘤浸润免疫细胞的丰度^[16, 17]^。TIMER数据库包括来自癌症TCGA的32种癌症类型的10, 897个样本，用于估计免疫浸润的丰度^[18]^。我们通过基因模块分析了不同类型癌症中的RTN1表达以及RTN1表达与免疫浸润丰度的相关性，包括B细胞、CD4^+^ T细胞、CD8^+^ T细胞、中性粒细胞、巨噬细胞和树突状细胞。针对肿瘤纯度的基因表达水平显示在最左侧的面板上。

### 1.4 人类蛋白质图谱(Human Protein Atlas, HPA)

人类蛋白质图谱(https://www.proteinatlas.org/)于2003年启动，利用多种组学技术绘制细胞、组织和器官中的所有人类蛋白质图谱，包括基于抗体的成像、基于质谱仪的蛋白质组学、转录组学和系统生物学。HPA由组织、单细胞类型、病理、血液、脑和细胞图谱组成。计算机检索HPA病理图谱中的“RTN1”(“肺癌”为癌类型)。然后，我们检查了每一幅图像的细节，并选择了LUAD中具有代表性的癌症和邻近样本的图像。

### 1.5 基因集富集分析

从TCGA下载516例LUAD患者的转录组数据，并根据RTN1表达的中值将RTN1表达高或低分组。GSEA检测到两组中排名靠前的基因富集的通路。在每次分析中，基因组排列设置为1, 000。

### 1.6 统计学分析

本研究统计学分析均通过在线网站TIMER 2.0(http://timer.comp-genomics.org/)完成。使用*Wilcoxon*检验分析两组样本的差异。使用*Pearson*卡方检验分析RTN1表达与临床特征变量之间的关联。*Cox*比例风险回归模型用于单变量和多变量分析。统计学差异设定为*P* < 0.05。

## 2 结果

### 2.1 RTN1表达高低与患者临床病理特征之间的关系

我们的分析发现，RTN1的表达水平与肺腺癌患者的生命状态、年龄、性别、吸烟史、肿瘤原发灶-淋巴结-转移(Tumor Node Metastasis, TNM)分期、肿瘤大小、淋巴结转移和远处转移等临床病理特征之间存在关联。根据RTN1 mRNA水平的中值，258例患者被归类为RTN1高表达，258例患者被归类为RTN1低表达。如表 1所示，RTN1表达与肺腺癌患者的生命状态(*P*=0.003)和性别(*P* < 0.001)显著相关。RTN1表达与其他临床病理因素之间无显著相关性，包括年龄(*P*=0.222)、吸烟史(*P*=0.068)、TNM分期(*P*=0.160)、肿瘤大小(*P*=0.162)、淋巴结转移(*P*=0.166)和远处转移(*P*=0.640)。

**表 1:** RTN1高低表达肺腺癌患者临床基本信息 Analysis of clinical data of lung adenocarcinoma patients with high and low expression of RTN1

Characteristic		Expression of RTN1		*P*
		High (*n*=258)	Low (*n*=258)	
Status	Alive	181 (70.1%)	148 (57.3%)	0.003
	Dead	77 (29.8%)	110 (42.6%)	
Age (yr)	≤60	68 (26.3%)	89 (34.5%)	0.065
	> 60	176 (68.2%)	158 (61.2%)	
Gender	Female	163 (63.1%)	115 (44.5%)	< 0.001
	Male	95 (36.8%)	143 (55.4%)	
Smoking status	Never	45 (17.4%)	30 (11.6%)	0.068
	Ever	204 (79.1%)	223 (86.4%)	
pTNM_stage	I/Ⅱ	212 (82.1%)	186 (72.0%)	0.160
	Ⅲ/Ⅳ	43 (16.7%)	67 (30.0%)	
T stage	T1/T2	226 (87.6%)	221 (85.7%)	0.162
	T3/T4	30 (11.6%)	36 (14.0%)	
N stage	N0	173 (67.0%)	159 (61.6%)	0.166
	N1/N2/N3	76 (29.4%)	96 (37.2%)	
M stage	M0	169 (70.3%)	178 (69.0%)	0.640
	M1	10 (6.2%)	13 (5.0%)	

### 2.2 RTN1在肺腺癌中表达情况及预后分析

为了研究肿瘤和正常组织之间RTN1表达水平的差异，我们使用TIMER 2.0和GEPIA 2分析RNA-seq数据。TIMER 2.0，将TCGA肿瘤组织与正常组织匹配，GEPIA 2，将TCGA肿瘤组织与GTEx数据匹配。应用TIMER 2.0分析正常组织样本和LUAD组织样本，结果(图 1A)显示LUAD组织中RTN1的mRNA水平显着低于正常组织。我们还发现，RTN1在大多数实体癌中是较低的，包括结肠腺癌、肺鳞癌、大肠腺癌和甲状腺癌。应用GEPIA 2的分析结果也证实了RTN1在LUAD组织中低表达(图 1B)。这些数据表明，LUAD患者的RTN1表达降低。同时为了研究RTN1在LUAD中的预后价值，我们使用GEPIA 2中的*Kaplan*-*Meier*生存分析工具分析了TCGA的预后数据。其结果表明(图 1C)，RTN1的高表达与患者更长的OS显著相关(HR=0.6, *P*=0.000)。

**图 1:**
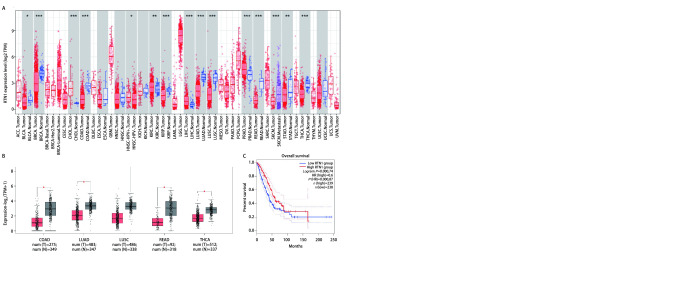
RTN1在LUAD中表达与预后情况。A: TIMER数据库预测RTN1在肺腺癌组织表达情况；B: GEPIA2数据库预测RTN1在肺腺癌组织表达情况；C: RTN1表达与肺腺癌患者预后的关系。 The expression level and prognostic value of RTN1 in patients with lung adenocarcinoma. A: Increased or decreased expression of RTN1 in cancers compared with adjacent normal tissue in TIMER database; B: Decreased expression of RTN1 in cancers compared with adjacent normal tissue in GEPIA database; C: The relationship between RTN1 expression and prognosis of patients with lung adenocarcinoma; LUAD: lung adenocarcinoma.

### 2.3 GO及KEGG富集分析RTN1调节相关通路

对TCGA数据库中肺腺癌患者的测序数据分析，取logFC < 1及FDR < 0.05做差异分析，我们发现共有245个基因上调，23个基因下调。对这些差异表达基因通过GO及KEGG富集分析后，我们发现其可能参与吞噬体的形成、造血细胞形成和细胞黏附的过程，且在GO富集分析中可以发现RTN1在T细胞活化起着重要作用，这提示我们该基因可能可以参与免疫微环境的调节(图 2A，图 2B)。

**图 2:**
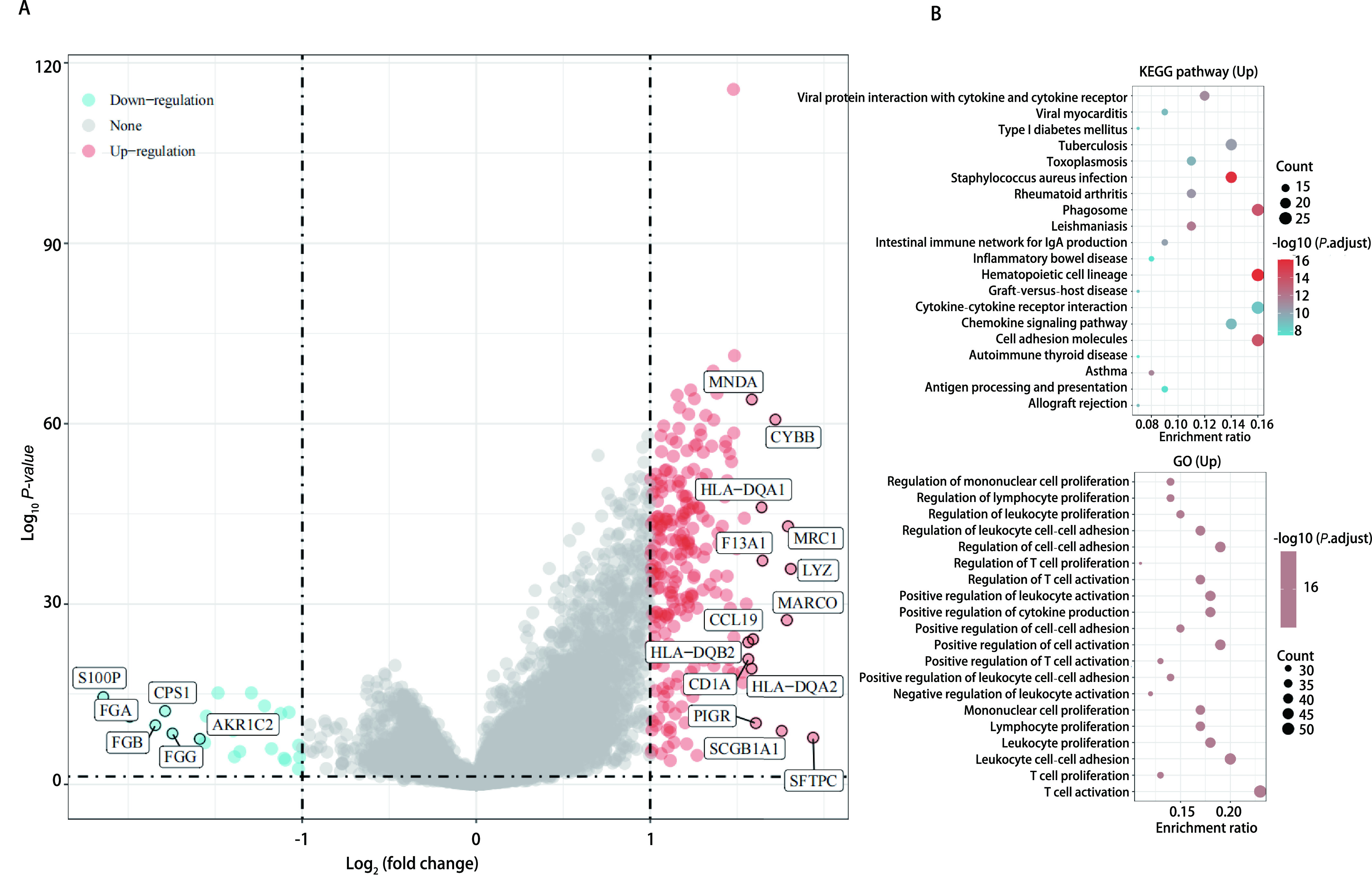
RTN1高表达患者基因通路富集分析。A：火山图；B：KEGG及GO富集分析。 GSEA pathways enriched in samples with high RTU1 expression. A: Volcanic map; B: KEGG and GO analysis.

### 2.4 LUAD中与RTN1共表达的基因分析

为了进一步研究RTN1在LUAD中的抑癌作用，我们使用cBioPortal与TCGA数据来分析与*RTN1*共表达正相关的基因。我们选择了与RTN1最显著相关的6个基因(图 3A-图 3F)。我们使用TIMER进一步验证了RTN1与这些基因之间的相关性。结果显示，*RTN1*与*BTK*、*CD4*、*ECSF1R*、*MNDA*、*NCKAP1L*和*SNX20*显著相关(图 3G-图 3L)。

**图 3:**
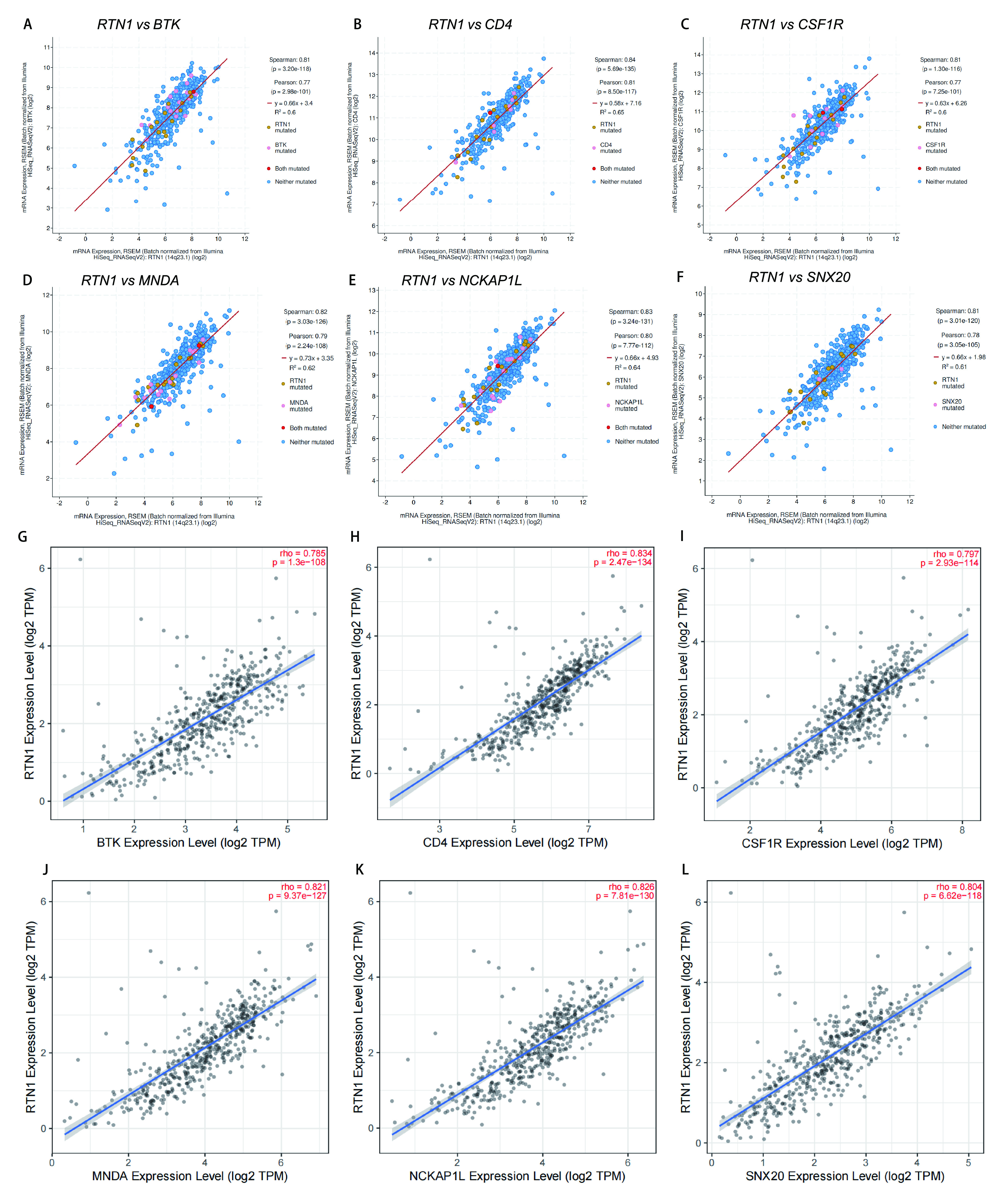
LUAD中RTN1的共表达基因。A-F: cBioPortal数据库肺腺癌队列鉴定与RTN1共表达的基因；G-L：TIMER数据库肺腺癌队列鉴定与RTN1共表达的基因。 Co-expressed genes of RTN1 in LUAD. A-F: The genes co-expressed with RTN1 in LUAD were assessed in cBioPortal database; G-L: The genes co-expressed with RTN1 in LUAD were assessed in the TIMER database.

### 2.5 *RTN1*及其共表达基因对肺癌组织免疫相关细胞浸润水平的影响分析

我们使用TISCH数据集在单细胞水平上(EMTAB 6149和GSE131907)研究了LUAD肿瘤微环境中RTN1的表达。在EMTAB 6149中，RTN1主要在免疫细胞中表达，包括B细胞、浆细胞、CD8 T ex细胞、CD8 T细胞、CD4 Tconv细胞、单/巨噬细胞、肥大细胞和调节性T细胞。RTN1在恶性细胞中轻度表达；然而，它几乎不在内皮细胞、成纤维细胞和肺泡细胞中表达。在GSE131907中，RTN1主要在B细胞、CD4 Tconv细胞、浆细胞、CD8 T细胞、CD8 Tex细胞、树突细胞、单/巨噬细胞和肥大细胞中表达，而在内皮细胞、成纤维细胞、上皮细胞和少突胶质细胞。此外，我们使用TIMER数据库来探索RTN1表达与免疫细胞浸润之间的关系。TIMER显示RTN1表达与几种类型的浸润免疫细胞呈正相关，包括B细胞、CD8^+^ T细胞、CD4^+^ T细胞、巨噬细胞、中性粒细胞和树突状细胞(图 4)。

**图 4:**
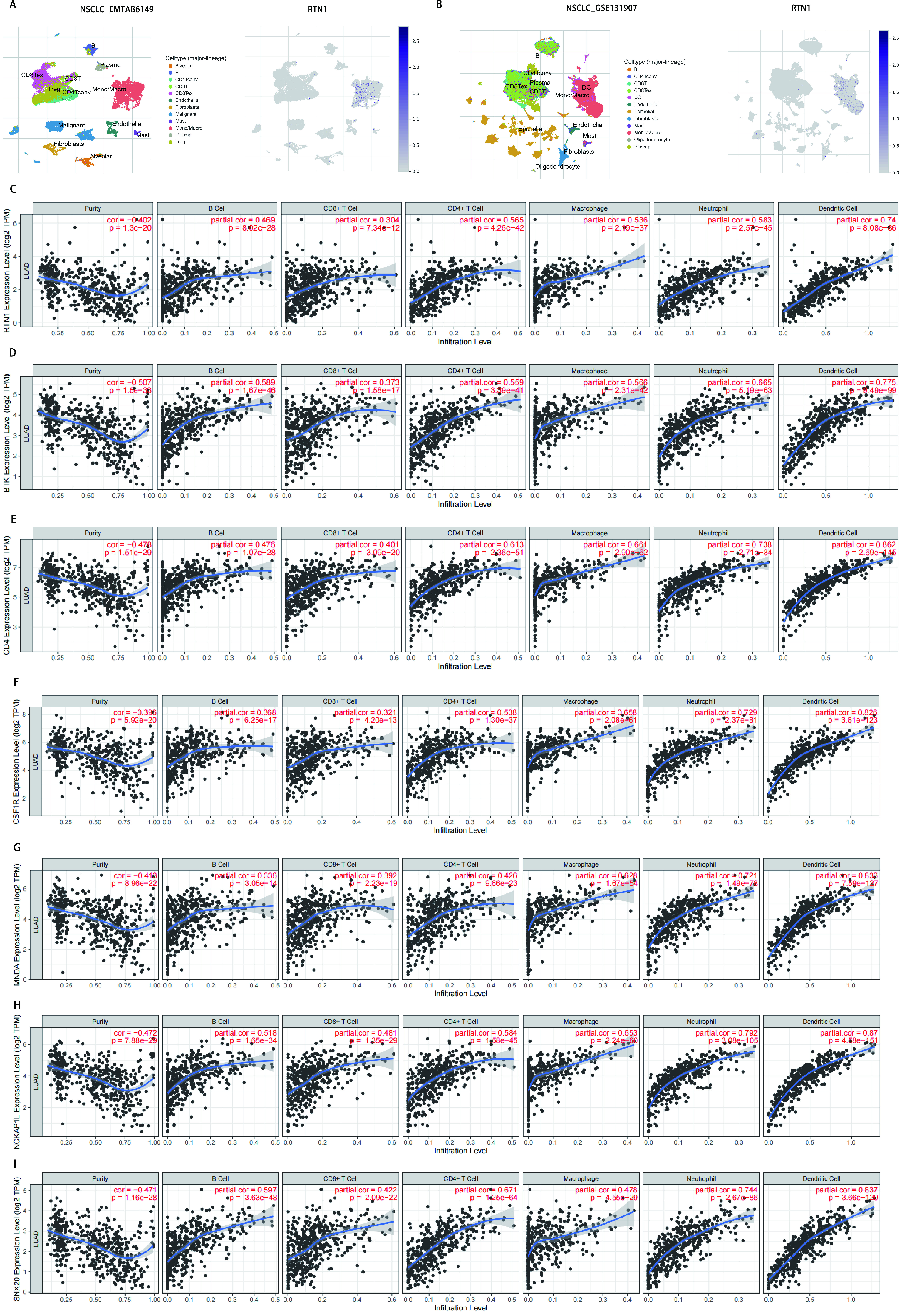
TCGA数据库肺腺癌队列RTN1及其共表达基因与肿瘤浸润免疫细胞的相关性。A，B：EMTAB 6149和GSE 131907数据集中LUAD微环境中RTN1的单细胞降维分群分析；C-I：数据库肺腺癌队列中RTN1与BTK、CD4、ECSF1R、MNDA、NCKAP1L和SNX20的表达与肿瘤浸润免疫的相关性分析。 Correlation of RTN1 and its co-expressed genes with tumor-infiltrating immune cell in LUAD. A, B: Single-cell distribution of RTN1 in the LUAD microenvironment in EMTAB 6149 and GSE 131907 datasets; C-I: Correlation of RTN1、BTK、CD4、ECSF1R、MNDA、NCKAP1L and SNX20 expression with tumor-infiltrating- immune in LUAD.

我们发现RTN1与几种免疫细胞之间存在显著的正相关关系(图 5)。CIBERSORT结果显示，RTN1高表达组CD4^+^ T细胞、肥大细胞、单核细胞、髓样树突状细胞和M1型巨噬细胞显著上调(图 5)，提示LRMP可能与免疫检查点有关。为进一步研究RTN1与免疫检查点之间的关系，我们将12例LUAD患者分为两组，比较两组免疫检查点的表达水平。G1组RTN1高表达，而G2组RTN1低表达。

**图 5:**
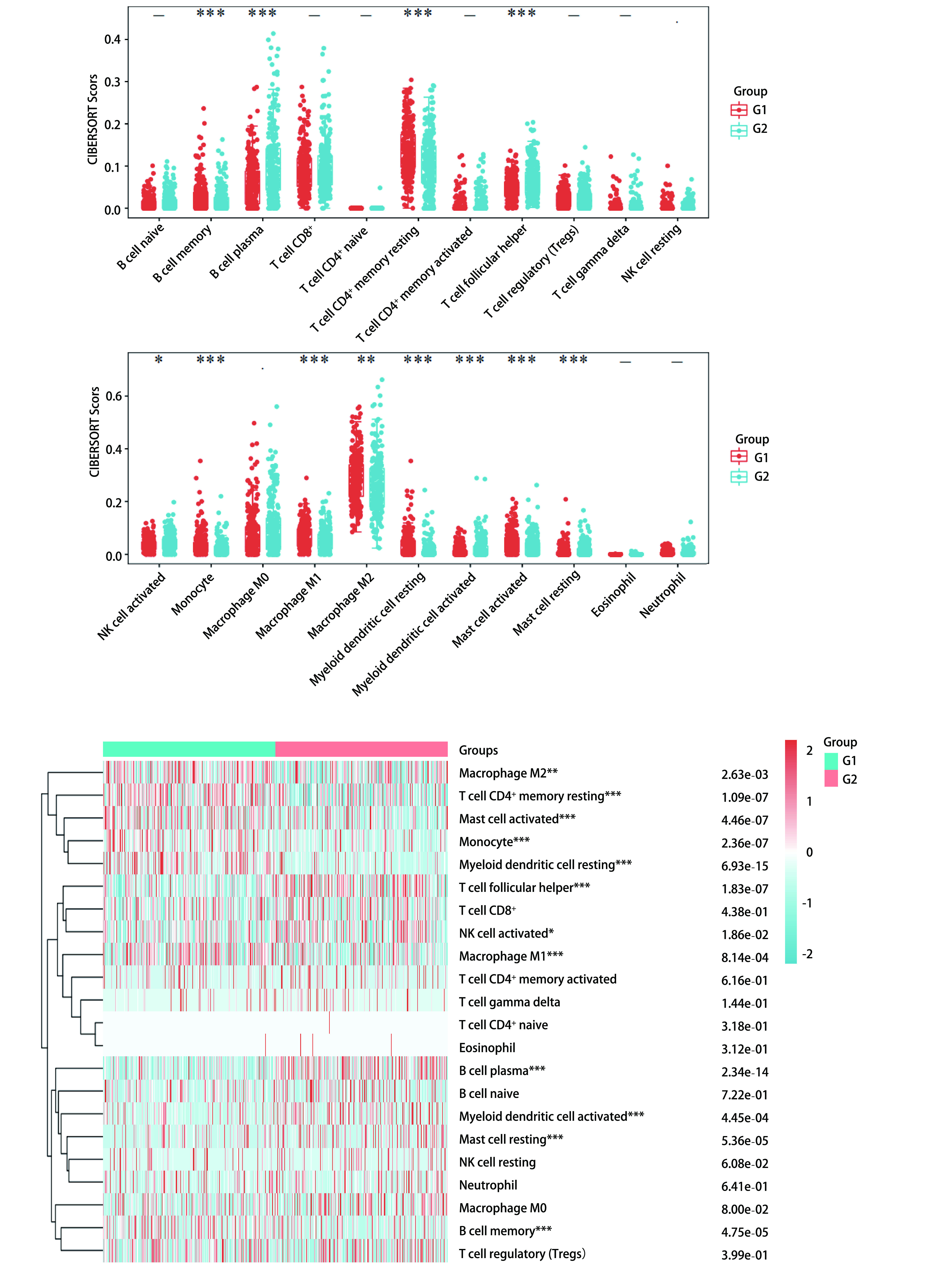
RTN1不同表达分组间的肺腺癌患者免疫微环境差异。G1：高表达; G2：低表达。 Changes of immune microenvironment in lung adenocarcinoma patients with different expression of RTN1. G1: high expression of RTN1; G2: low expression of RTN1.

### 2.6 RTN1表达与免疫检查点密切相关

高低表达患者并将其分别G1(高表达)组及G2(低表达)组，使用TIMER 2.0数据库分析发现，RTN1表达与常见免疫检查点CD274、CTLA4、HAVCR2、LAG3、PDCD1、PDCD1LG2、TIGIT、SIGLEC15等的表达显著相关(图 6)，提示我们RTN1可能与免疫检查点密切相关，且有望在免疫治疗中发挥作用。

**图 6:**
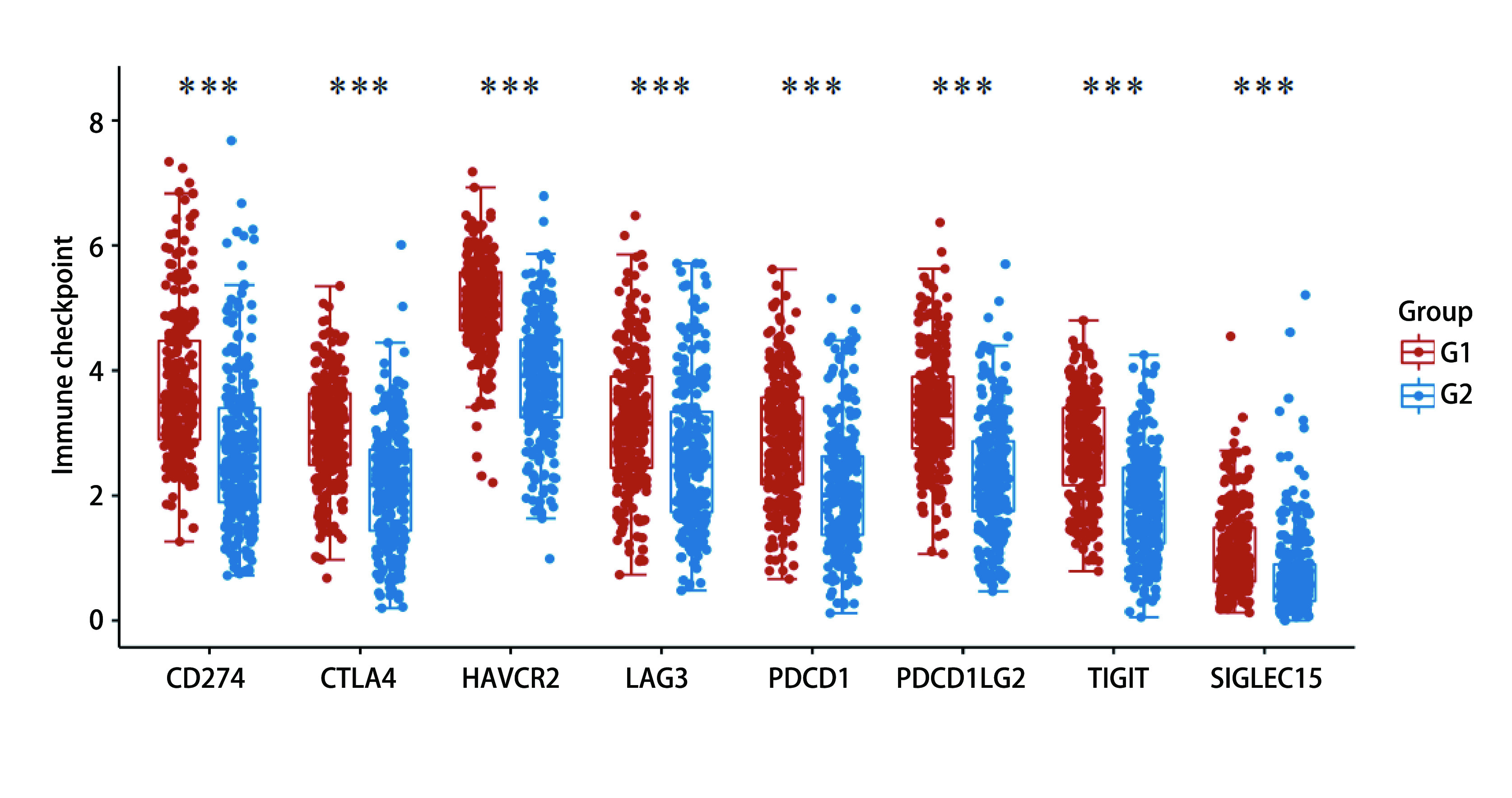
RTN1表达量对免疫检查点表达的影响 The influence of RTN1 on the expressions of immune checkpoints

## 3 讨论

基因表达异常与肺腺癌的发生和发展密切相关，并预示着肺腺癌患者的预后。然而，肺腺癌和其肿瘤微环境相互作用的分子机制尚不清楚。在本研究中，我们证明RTN1在LUAD组织中的表达显著降低，并且与LUAD患者的生命状态、性别等有关。此外，RTN1的低表达与预后和总存活率有关。这些结果提示*RTN1*可能是一种肿瘤抑制基因，参与了肺腺癌的进展。

*RTN1*是一种与内质网相关的网状体编码基因。*RTN1*在神经内分泌细胞的膜运输或神经内分泌分泌中起关键作用，同时RTN1作为神经系统疾病和癌症的潜在诊断/治疗标志物。然而在肺腺癌患者中RTN1的表达情况及其对免疫微环境影响均未有报道。本研究旨在使用公共数据库和生物信息学网络工具研究RTN1在肺癌中的表达及其与肺癌中免疫浸润和存活的相关性。在本项研究中，我们发现RTN1在多种癌症中异常表达，表明它可能在肿瘤的发生中发挥作用。在LUAD中，我们发现RTN1在肿瘤组织中的表达明显低于在正常组织中的表达，提示RTN1可能参与了LUAD的肿瘤抑制作用。此外，本研究还验证了RTN1在LUAD细胞系和肿瘤组织中的表达降低。

为了进一步探讨RTN1在LUAD肿瘤发生中的分子功能和可能的机制，我们进行了GSEA分析，以探索在RTN1高表达的样本中丰富的途径。共选择了15条与细胞黏附、免疫和炎症反应相关的通路。在KEGG富集分析中发现其与“Hematopoietic cell lineage”信号通路可能有密切关系，同时GO富集分析发现其与T细胞的活化可能有密切关系。

我们通过TCGA和TIMER数据库双重验证，发现*RTN1*与*BTK*、*CD4*、*ECSF1R*、*MNDA*、*NCKAP1L*和*SNX20*显著相关。进一步对其共表达相关基因进行分析后，我们发现RTN1表达改变时也会引起免疫效应细胞如CD4^+^ T细胞，CD8^+^ T细胞比例的升高^[19, 20]^。免疫微环境中效应细胞比例的升高往往提示着患者的较好预后。最后我们发现RTN1表达与常见免疫检查点CD274、CTLA4、HAVCR2、LAG3、PDCD1、PDCD1LG2、TIGIT、SIGLEC15等的表达显著相关。

尽管我们的研究结果表明RTN1与免疫浸润相关并可作为LUAD的预后生物标志物，但该研究仍有一些局限性。首先，我们的研究基于有限的数据样本，可能存在一定的偏差。其次，该发现是否适用于所有具有不同分子改变的LUAD患者仍有待证实。综上所述，*RTN1*可能作为一种肿瘤抑制基因并表明更好的预后。此外，*RTN1*与可能参与LUAD中免疫治疗反应的免疫浸润有关。需要进一步的研究来验证这些发现。
